# Supplementary figures and images for: Bovine pain scale: A novel tool for pain assessment in cattle undergoing surgery in the hospital setting
Source: PLoS One. 2025 May 23;20(5):e0323710. doi: 10.1371/journal.pone.0323710 (PMC12101770; doi:10.1371/journal.pone.0323710)

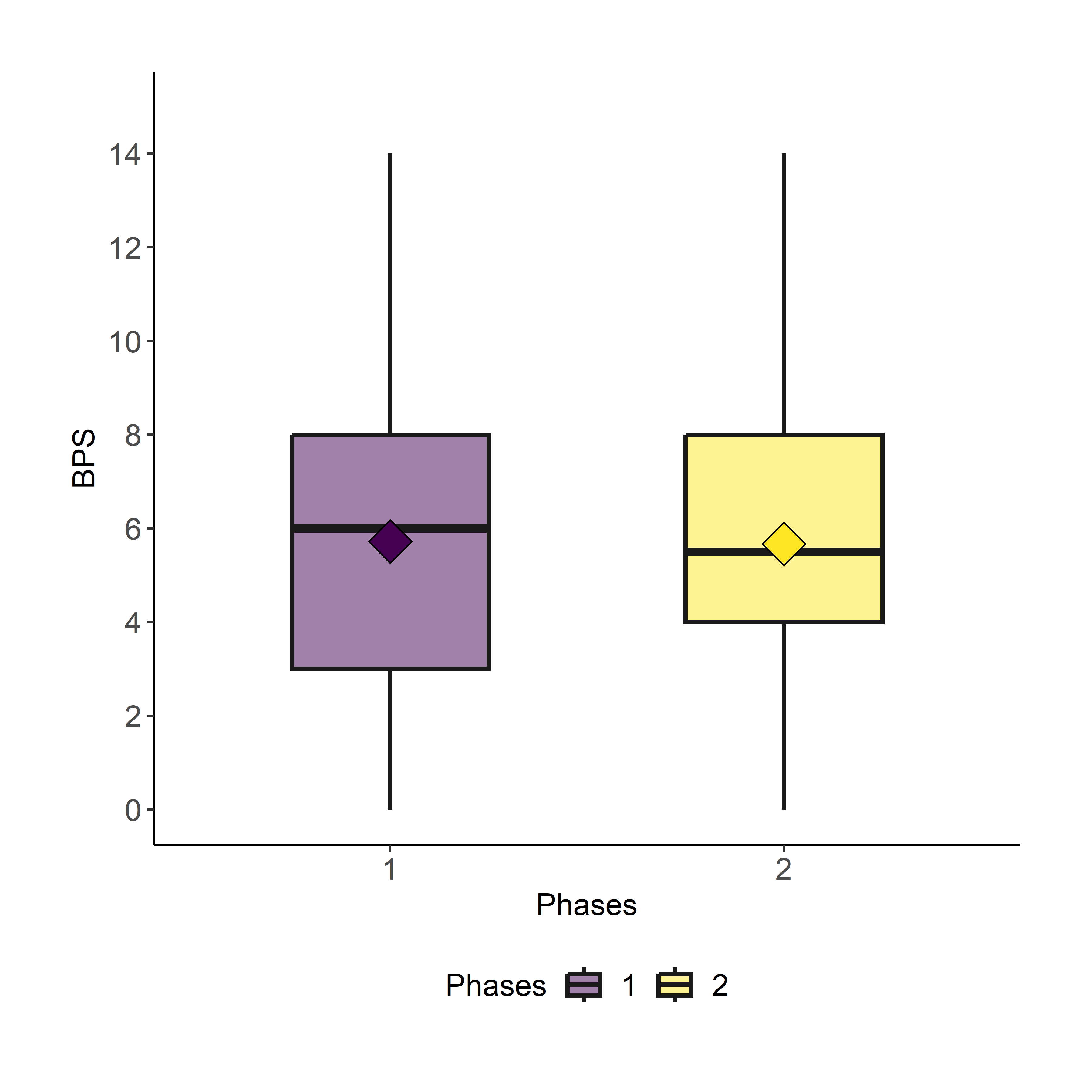

Supplement: S1 Fig — (TIF) [file pone.0323710.s001.tif]

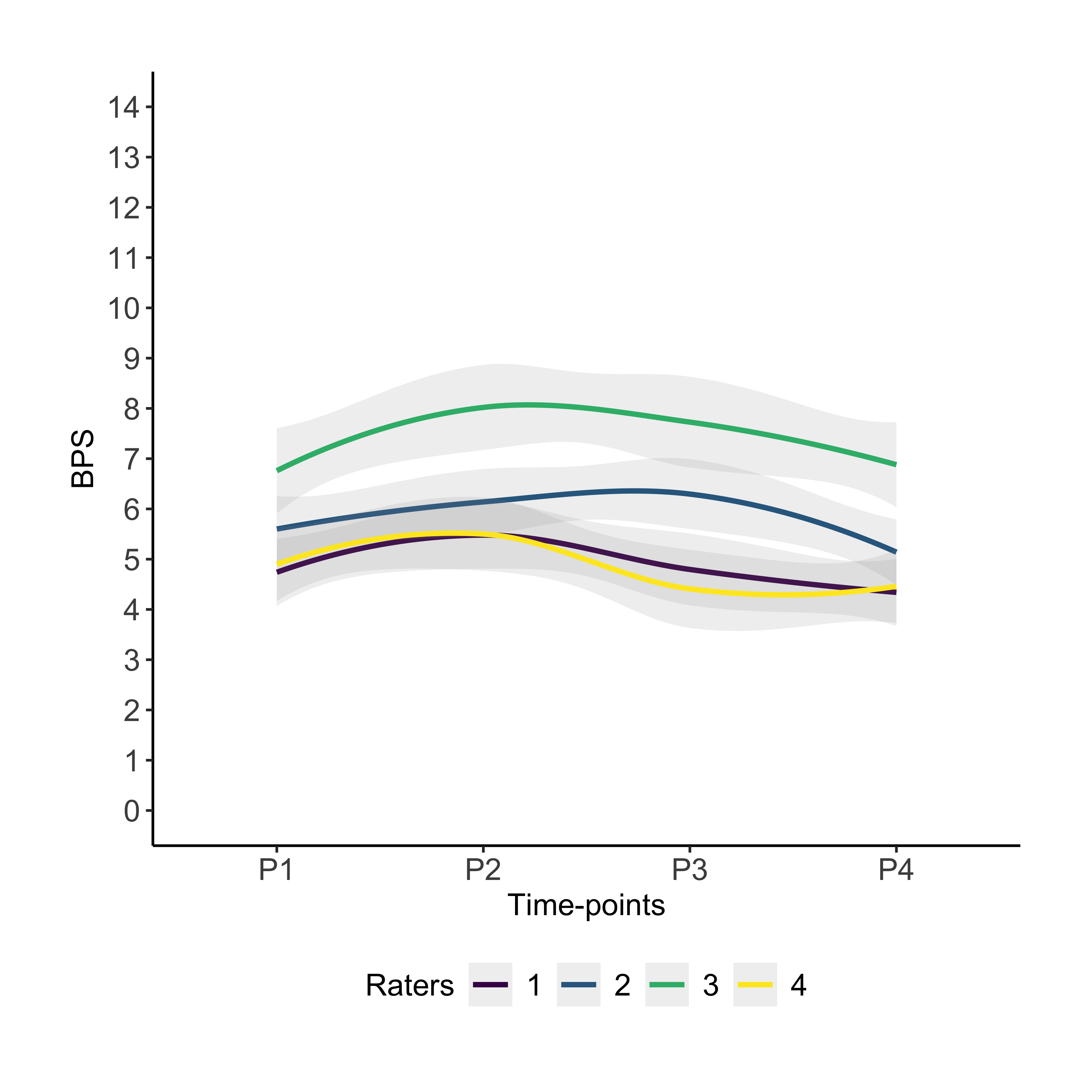

Supplement: S2 Fig — Time-points: P1, preoperatively, immediately prior to administration of sedatives; P2, 2–6 hours after the end of surgery; P3, 1 hour after the administration of analgesic intervention if required; P4, 24 hours after surgery; were assessed. (TIF) [file pone.0323710.s002.tif]
