# Supplementary material for: Bovine pain scale: A novel tool for pain assessment in cattle undergoing surgery in the hospital setting
Source: PLoS One. 2025 May 23;20(5):e0323710. doi: 10.1371/journal.pone.0323710 (PMC12101770; doi:10.1371/journal.pone.0323710)
Supplement: S1 Table — (DOCX) [file pone.0323710.s003.docx]

**S1 Table.** **Anaesthetic protocols of animals included in the Pain Group (n = 25).**

| **Animal** | **Surgery type** | **Body weight** | **Anaesthetic protocol** |
| --- | --- | --- | --- |
| 3 | Theloscopy front right teat | 890 Kg | Sedation: Xylazine 0.05mg/kg IV; Buthorphanol 0.025mg/kg IV; Ketamine 0.1mg/kg IV. Local block: Lidocaine 2% - 20ml ring block of front right teat, and 8ml in the teat cistern. Analgesics: Flunixin Meglumine 1mg/kg IV. Rescue Analgesia: Buthorfanol 0.05mg/kg IM |
| 5 | Phlebectomy of varicose vein | 695 kg | Sedation: Acepromazine 0.02mg/kg IV; GGE 5% 30mg/kg IV; Ketamine 0.4mg/kg IV; Butorphanol 0.07mg/kg IV. Maintenance: Triple drip 2.5ml/kg/h IV (GGE 5% 600ml; ketamine 600mg; xylazine 20mg). Local block: Lidocaine 2% - 25ml - ring block of left front teat. Analgesics: Flunixin meglumine 1mg/kg IV |
| 7 | Mandibular fracture | 920 kg | Sedation/PAM: GGE 5% 3000mg IV; Butorphanol 19mg IV; Xylazine 6mg IV. Induction: Ketamine 2000mg IV; Diazepan 50mg IV. Maintenance: Isoflurane. Local block: Bupivacaine 0.5% - 20ml in the left mandibular nerve. Analgesics: Butorphanol 19mg IV. Rescue Analgesia: Buthorfanol 0.05mg/kg IM |
| 8 | Left metacarpal fracture | 340 kg | Sedation/PAM: Acepromazine 5 mg IV; Morphin 40mg IV; Xylazine 10mg IV. Induction: Ketamine 1000mg IV; Diazepan 40mg IV. Maintenance: Isoflurane. Analgesics: Meloxicam; Ketamina 150mg IV. Rescue Analgesia: Buthorfanol 0.05mg/kg IV |
| 9 | Lumbar mass removal | 718 kg | Sedation: Ketamine 60mg IV; Xylazine 30mg IV. Analgesics: Butorphanol 15mg IV |
| 10 | Partial teat amputation | 608 kg | Sedation: Acepromazine 20mg IM. Local block: Lidocaine 2% - 25ml ring block in the teat Q2 and 6ml in the teat cistern. Analgesics: Flunixin 1.1mg/kg IM. |
| 11 | Theloscopy front right teat | 520 kg | Sedation: Xylazine 25mg IV; Ketamine 70mg IV. Induction: GGE 5% 500ml IV; Ketamine 500mg IV. Local block: Lidocaine 2% - 23 ml ring block on the front right teat and 7ml in the teat cistern. Analgesics: Butorphanol 18mg IV; Flunixin Meglumine 650mg IM. Rescue Analgesia: Buthorfanol 0.05mg/kg IM |
| 12 | Mass removal | 800 kg | Local block: Lidocaine 2% - 40ml block around mass. Analgesics: Ketoprofen 24ml IM. Rescue Analgesia: Buthorfanol 0.05mg/kg IM |
| 13 | Rumenotomy | 650 kg | Local block: Paravertebral block - Lidocaine 2% - 35ml. Analgesics: Flunexin Meglumine 1.1mg/kg IV. Rescue Analgesia: Buthorfanol 0.05mg/kg IV |
| 14 | Left posterior hock hygroma | 610 kg | Sedation/PAM: GGE 5% 3500mg IV; Butorphanol 18.3mg IV; Acepromazine 4.6 mg IV. Induction: Diazepan 30.5mg IV; Ketamine 150mg IV. Maintenance: Isoflurane. Local block: Epiural lumbosacral with Morphine 0.01 mg/kg and Bupivacaine 0.06mg/kg. Analgesics: Butorphanol 18.3mg IV; Ketamina 500mg IV. Rescue Analgesia: Buthorfanol 0.05mg/kg IM |
| 15 | Rumenotomy | 690 kg | Local block: Paravertebral block lidocaine 2% - 30ml. Analgesics: Butorphanol 30mg SC. Rescue Analgesia: Buthorfanol 0.05mg/kg IV |
| 16 | Caesarean section | 754 kg | Local block: Paravertebral block lidocaine 2% - 120ml. Rescue Analgesia: Buthorfanol 0.05mg/kg IV |
| 17 | Theloscopy hind right teat | 607 Kg | Sedation: Acepromazine 9mg IV. Local block: Lidocaine 2% - 10ml ring block of hind right teat. Analgesics: Flunixin 1.1mg/kg IM. Rescue Analgesia: Buthorfanol 0.05mg/kg IM |
| 19 | Enucleation | 782 kg | Sedation: Acepromazine 20mg IV. Local block: Lidocaíne 2% - 30ml peterson block + palpebral block. Rescue Analgesia: Buthorfanol 0.05mg/kg IM |
| 21 | Cranial cruciate ligament repair | 774 kg | Sedation/PAM: Acepromazine 15.5mg IV; Xylazine 20mg IV; Buthorphanol 35mg IV. Induction: Ketamine 2202mg IV; Diazepam 40mg IV; Ketamine 500mg IV. Maintenance: Isoflurane. GGE 5% 1l IV. Local block: Epidural lumbosacral morphine 0.1ml/kg. Analgesics: Morphine 77mg IV. Rescue Analgesia: No rescue due to morphine epidural |
| 22 | Abomasopexy | 705 kg | Local block: Paravertebral block, Lidocaine 2% - 140ml. Analgesics: Flunixin 1.1mg/kg IM. Rescue Analgesia: Buthorfanol 0.05mg/kg IV |
| 23 | Laparoscopy and omentopexy | 555 kg | Local block: Paravertebral and ring block lidocaine 2% - 155ml. Rescue Analgesia: Buthorfanol 0.05mg/kg IM |
| 24 | Laparoscopy and ovariectomy | 764 kg | Local block: Paravertebral and ring block lidocaine 2% - 145ml. Analgesics: Flunixin Meglumine 1.1mg/kg IM. Rescue Analgesia: Buthorfanol 0.05mg/kg IM |
| 25 | Rumenotomy | 680 kg | Local block: Paravertebral and ring block lidocaine 2% - 40ml. Rescue Analgesia: Buthorfanol 0.05mg/kg IV |
| 26 | Enucleation | 800 kg | Sedation: Acepromazine 35mg IV. Local block: Peterson block lidocaine 2% - 55ml; line block 45ml, auricular block 15ml, palpebral splash block 10ml. Rescue Analgesia: Buthorfanol 0.05mg/kg IM |
| 27 | Abomasopexy | 825 kg | Local block: Paravertebral proximal block lidocaine 2% - 135ml. Rescue Analgesia: Buthorfanol 0.05mg/kg IV |
| 28 | Stifle / meniscus repair | 340 kg | Sedation: Xylazine 10mg IV; Ketamina 20mg IV. Local block: Epidural lidocaine 2% - 66ml. Analgesics: Butorphanol 5mg IV. Rescue Analgesia: Buthorfanol 0.05mg/kg IV |
| 29 | Caesarean | 698 kg | Local block: Paravertebral proximal block lidocaine 2% - 105ml. Analgesics: Meloxicam 16ml IM. Rescue Analgesia: Buthorfanol 0.05mg/kg / IM |
| 30 | Rumenotomy | 650 kg | Sedation: Acepromazine 20mg IM. Local block: Paravertebral proximal block lidocaine 2% - 210ml. Analgesics: Meloxicam 16ml IM. Rescue Analgesia: Buthorfanol 0.05mg/kg IM |
| 31 | Laparoscopy and abomasopexy | 660 kg | Sedation: Acepromazine 20mg IV; Xylazine 20mg IV; Ketamina 870mg IV + 15mg IV +150mg IV. Analgesics: Butorphanol 30mg IV + 10mg IV; Flunixin Meglumine 13ml IM. Rescue Analgesia: Buthorfanol 0.05mg/kg IV |

Legend: IV – intravenous route. IM – intramuscular route. Mg – miligrama. Kg - kilogram. PAM – Pre-Anaesthetic Medication
