# Supplementary material for: Bovine pain scale: A novel tool for pain assessment in cattle undergoing surgery in the hospital setting
Source: PLoS One. 2025 May 23;20(5):e0323710. doi: 10.1371/journal.pone.0323710 (PMC12101770; doi:10.1371/journal.pone.0323710)
Supplement: S3 Supplementary material — (DOCX) [file pone.0323710.s006.docx]

**Training Manual**

***Be familiar with the tool***

1) Please read carefully the Bovine Pain Scale (9 items - facial expression, appetite, posture when standing, posture when lying down, miscellaneous behaviours, limb movement/condition, interactive behaviour - with the environment, response to approach, activity, and locomotion; each item includes 3 descriptive levels) provided in the table 1.

**Training**

2) Watch the behaviours of each item in Bovine Pain Scale (Table 1), where every score or behaviour is attached to a link for its respective video (google drive folder). We highly suggest the raters watch the videos with full screen, and high resolution (when you open the video, in the right inferior corner, you can find the *Settings* symbol, click there, after choosing: *Quality*, and you will see the options from auto - 360p to 1080p HD).

3) If you have questions regarding any of these behaviours, please contact the authors for clarification before the video analysis. 

**Table 1. Bovine Pain Scale**

| ITEM | VARIABLE | VIDEO LINK |
| --- | --- | --- |
| Appetite | (0) Normorexia and/or rumination | <https://drive.google.com/file/d/10GQFw2rJblzCh_sY9RAlsoiuzMFpXmNu/view?usp=sharing> |
|  | (1) Hyporexia | <https://drive.google.com/file/d/1kFOCPeVLirBAM_yDxF5tdTPJYrk1tBx6/view?usp=sharing> |
|  | (2) Anorexia | <https://drive.google.com/file/d/1Uw4wVDn9pBanYKhlPJO5PZvoezdP9tSG/view?usp=sharing> |
| Posture when standing | Arching the back (except when standing up or urinating) | <https://drive.google.com/file/d/1C9MxvvRbOA9_4tqqgkoJO1myDcb8wKff/view?usp=sharing> |
|  | Hind limbs extended caudally (observe from the side) | <https://drive.google.com/file/d/1IPppDDMWPBCl92mqMj295Y0mfA17natl/view?usp=sharing> |
|  | Top of the head below the line of spinal column (if not eating) | <https://drive.google.com/file/d/189knbkOoNQ2WIrTWVtl9yo41OMEHp8tr/view?usp=sharing> |
|  | (0) All of the above-described behaviours are absent |  |
|  | (1) Presence of 1 of the above-described behaviours |  |
|  | (2) Presence of 2 or more of the above-described behaviours |  |
| Posture when lying down | Ventral recumbency with full or partial extension of one or both hind limbs | <https://drive.google.com/file/d/16vEf4ajMnrxpn6GpjbJAuDvcXMw0UYga/view?usp=sharing> |
|  | Head on/close to the ground | <https://drive.google.com/file/d/1ZnNmlDGxyEj6vs5UvrSIrGa7zQ6tpiwg/view?usp=sharing> |
|  | Extending the neck and body forward when in ventral recumbency | <https://drive.google.com/file/d/1g6c0NXEzQdr5ME9HFXMj8uZjbfrtdz-9/view?usp=sharing> |
|  | (0) All of the above behaviours are absent |  |
|  | (1) Presence of 1 of the above-described behaviours |  |
|  | (2) Presence of 2 or more of the above-described behaviours |  |
| Miscellaneous behaviours 1 | Groaning | <https://drive.google.com/file/d/1v-OrqbZz5kNGLdsNYcIb7kQQqvQn41TW/view?usp=sharing> |
|  | Attention towards the painful area | <https://drive.google.com/file/d/1prWKOGlxJGF6hahCxFcX3HYptpCRF3W6/view?usp=sharing> |
|  | Licking the surgical wound | <https://drive.google.com/file/d/1WHz71jJ_ksnvH2p_Oz5x32LB0yrO2TLy/view?usp=sharing> |
|  | (0) All of the above behaviours are absent |  |
|  | (1) Presence of 1 of the above-described behaviours |  |
|  | (2) Presence of 2 or more of the above-described behaviours |  |
| Miscellaneous behaviours 2 | Lambs’ ears, ears rotated back and the pinna facing down | <https://drive.google.com/file/d/1p3ns-ZCN3XAbIUbWqdwDLruCmtBXi12S/view?usp=sharing> |
|  | Tense expression/strained appearance, furrows above the eyes and puckers above the nostrils | <https://drive.google.com/file/d/1lIZF7Iq_J4zjj9kGRliH_AzQBUQQ4tXy/view?usp=sharing> |
|  | Wagging the tail abruptly and repeatedly | <https://drive.google.com/file/d/1DTIsyEzO4giaWrjoDrvFcUUhH4XaFNFX/view?usp=sharing> |
|  | (0) All of the above behaviours are absent |  |
|  | (1) Presence of 1 of the above-described behaviours |  |
|  | (2) Presence of 2 or more of the above-described behaviours |  |
| Limb movement/condition | Lifting one foot of the ground | <https://drive.google.com/file/d/1Icsjdw1vS246h0cOhkFA1VrPFYyoq5P-/view?usp=sharing> |
|  | Kicking/foot stamping | <https://drive.google.com/file/d/16ewgpeMK5E5gLeuFTThuU09mENNUFKuE/view?usp=sharing> |
|  | Restlessness (pacing) | <https://drive.google.com/file/d/1JiiB0_5w1p7JIEcEU8MZDE9-eg5NboJ1/view?usp=sharing> |
|  | Weight shifting | <https://drive.google.com/file/d/1WbsrSPA-W7D8vgVP7MnvVD3S6ZA31N_B/view?usp=sharing> |
|  | (0) All of the above described behaviours are absent |  |
|  | (1) Presence of 1 of the above-described behaviours |  |
|  | (2) Presence of 2 or more of the above-described behaviours |  |
| Interactive behaviour  with the environment | (0) Active and attentive to environmental stimuli*. When near other animals, can interact with and/or accompany the group | <https://drive.google.com/file/d/1VHuYJmUrKfwbxkLOB2fd4599VU0GYJWz/view?usp=sharing> |
|  | (1) Apathetic, interacting little when stimulated. When near other animals might remain close to them | <https://drive.google.com/file/d/1tKLcV49KTEbkZOyey_TAn_uwZV0TJmLh/view?usp=sharing> |
|  | (2) Apathetic; not reacting to environmental stimuli. When near other animals may be isolated or not accompany them | <https://drive.google.com/file/d/1L9uPJIv4j4XQQHRC9jd6Q8e_6lNyzpu0/view?usp=sharing> |
| Response to approach | (0) Animal’s head up, ears forward, or may interrupt briefly ongoing activity (grooming, ruminating, etc.) | <https://drive.google.com/file/d/1aLPrHo5GujMGynqRkIwK0JEbsntu7cMc/view?usp=sharing> |
|  | (1) Animal’s ears not forward, orients by moving head in the direction of the observer | <https://drive.google.com/file/d/1tPYQ8pe6_xNEvA7BcoAar9LVP1Z3ut4_/view?usp=sharing> |
|  | (2) Animal’s ears back, head low, does not orient head toward the observer clapping hands (no head movement) | <https://drive.google.com/file/d/1HCtFEVwlRbLM-QjImP_NCkWsfKrzatzG/view?usp=sharing> |
| Activity and locomotion  *(if the animal is lying down or standing still stimulate it with clapping hands)* | (0) Moving normally. Walking with no obviously abnormal gait, or relaxed in ventral recumbency position (resting quietly), or standing still easily or eating or ruminating | <https://drive.google.com/file/d/1WClix5-3iOiLPjdfBGpd0VqUUfkt9e9y/view?usp=sharing> |
|  | (1) Walking with restriction, hunched back when moving or  short steps. May be agitated (constant changes in weight-bearing) or laying restlessness (difficult to find a comfortable position) | <https://drive.google.com/file/d/1HhdXy-TPuIZJQCGzkX7b-jwIdMm8-c_Z/view?usp=sharing> |
|  | (2) Reluctant to stand up, standing up with difficulty or not walking | <https://drive.google.com/file/d/1pzla6huHZ8wesiAURDmSs5nHrsNiFYjK/view?usp=sharing> |
