# Supplementary material for: Bovine pain scale: A novel tool for pain assessment in cattle undergoing surgery in the hospital setting
Source: PLoS One. 2025 May 23;20(5):e0323710. doi: 10.1371/journal.pone.0323710 (PMC12101770; doi:10.1371/journal.pone.0323710)
Supplement: S4 Supplementary material — (PDF) [file pone.0323710.s007.pdf]

# Main Study - Video Analysis Part 2 - Bovine Pain Scale Project (Week 1)

## 1. Welcome to the Week 1 - Video Analysis Part 2 of the Bovine Pain Scale Project!

The Main Study Part 2 consists of the analysis of 118 videos (6min/video = ±11.8hrs/videos) in two parts.
The Week 1 contains 20 videos for analysis. Please watch each video and answer the questionnaire.
The sound should be on during video assessment, and you can watch the videos as many times you need before scoring.

If the item in the Bovine Pain Scale is not visible in the video, please mark the option "not possible to score".
In cases of the checkbox questions, please tick each behaviour you see (can be more than one, or all them), or in case they are not present tick option "(0) All of the above-described behaviours are absent".

You can go back at any time, check and edit all your answers before finishing the survey.

* 1. Rater's name:

1 / 21 5%

Next

Powered by
SurveyMonkey
See how easy it is to create surveys and forms.

## Main Study - Video Analysis Part 1 - Bovine Pain Scale Project (Week 1)

### 2. Video 1

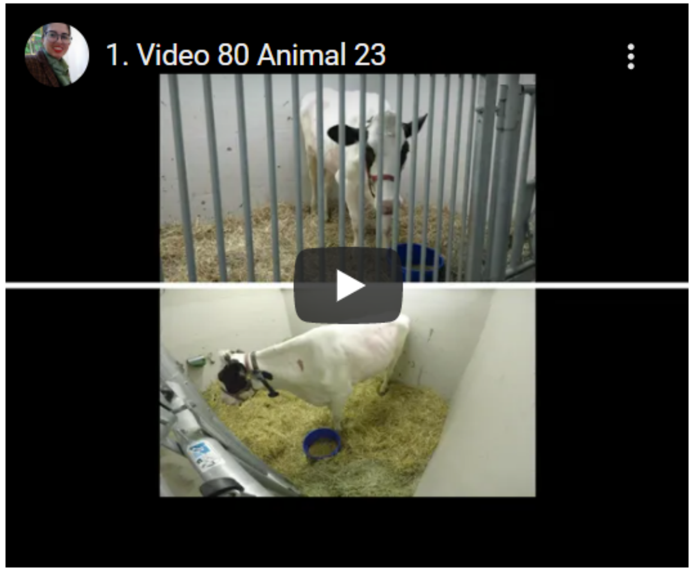


Please watch the following sample video, and then answer the survey.

Please select the video quality for the best possible (HD definition). By clicking on “YouTube” at the bottom of the video, you will be prompted to a new window where you may select to watch the video in full screen.

You can watch how many times you need.

* 1. Video 1. Item - Activity and locomotion

- ◯ (0) Moving normally. Walking with no obviously abnormal gait, or relaxed in ventral recumbency position (resting quietly), or standing still easily or eating or ruminating
- ◯ (1) Walking with restriction, hunched back when moving or short steps. May be agitated (constant changes in weight-bearing) or laying restlessness (difficult to find a comfortable position)
- ◯ (2) Reluctant to stand up, standing up with difficulty or not walking
- ◯ Not possible to score

* 2. Video 1. Item - Response to approach

- ◯ (0) Animal’s head up, ears forward, or may interrupt briefly ongoing activity (grooming, ruminating, etc.)
- ◯ (1) Animal’s ears not forward, orients by moving head in the direction of the observer
- ◯ (2) Animal’s ears back, head low, does not orient head toward the observer clapping hands (no head movement)
- ◯ Not possible to score

* 3. Video 1. Item - Interactive behaviour with the environment

**Tactile and/or visual and/or audible environmental stimuli in response to touching and/or to approach of people/or other animals and/or from environmental sounds (animals or people around or clapping hands).

- ◯ (0) Active and attentive to environmental stimuli**. When near other animals, can interact with and/or accompany the group
- ◯ (1) Apathetic, interacting little when stimulated. When near other animals might remain close to them
- ◯ (2) Apathetic; not reacting to environmental stimuli. When near other animals may be isolated or not accompany them

* 4. Video 1. Item - Limb movement/condition

- [ ] Lifting one foot of the ground
- [ ] Kicking/foot stamping
- [ ] Restlessness (pacing)
- [ ] Weight shifting
- [ ] (0) All of the above-described behaviours are absent

* 5. Video 1. Item - Miscellaneous behaviours 1

- [ ] Groaning
- [ ] Attention towards the painful area
- [ ] Licking the surgical wound
- [ ] (0) All of the above-described behaviours are absent

* 6. Video 1. Item - Posture when standing

- [ ] Arching the back (except when standing up or urinating)
- [ ] Hind limbs extended caudally (observe from the side)
- [ ] Top of the head below the line of spinal column (if not eating)
- [ ] (0) All of the above-described behaviours are absent

* 7. Video 1. Item - Appetite

- ◯ (0) Normorexia and/or rumination
- ◯ (1) Hyporexia
- ◯ (2) Anorexia
- ◯ Not possible to score

* 8. Video 1. Would you give rescue analgesia to this animal?

- ◯ Yes (1)
- ◯ No (0)

* 9. Video 1. Item - Miscellaneous behaviours 2

- ☐ Lambs' ears, ears rotated back and the pinna facing down
- ☐ Tense expression/strained appearance, furrows above the eyes and puckers above the nostrils
- ☐ Wagging the tail abruptly and repeatedly
- ☐ (0) All of the above-described behaviours are absent

* 10. Video 1. Item - Posture when lying down

- [ ] Ventral recumbency with full or partial extension of one or both hind limbs
- [ ] Head on/close to the ground
- [ ] Extending the neck and body forward when in ventral recumbency
- [ ] (0) All of the above-described behaviours are absent

* 11. Video 1. On a visual analogue scale (VAS) 1 to 100, how would you score the animal pain?

1 — 100

* 12. Video 1. How many times have you watched the video?

- ◯ 1 time
- ◯ 2 times
- ◯ 3 times
- ◯ 4 times
- ◯ 5 times
- ◯ More than 5 times

2 / 21 — 10%

Prev Next

Powered by
SurveyMonkey
See how easy it is to create surveys and forms.
