# Supplementary material for: Bovine pain scale: A novel tool for pain assessment in cattle undergoing surgery in the hospital setting
Source: PLoS One. 2025 May 23;20(5):e0323710. doi: 10.1371/journal.pone.0323710 (PMC12101770; doi:10.1371/journal.pone.0323710)
Supplement: S5 Supplementary material — (DOCX) [file pone.0323710.s008.docx]

The **Bovine Pain Scale** consists of nine items with inclusion of three descriptive levels

| **ITEM** | **VARIABLE** | **Score** |
| --- | --- | --- |
| **Appetite** | (0) Normorexia and/or rumination |  |
|  | (1) Hyporexia |  |
|  | (2) Anorexia |  |
| **Posture when standing** | Arching the back (except when standing up or urinating) |  |
|  | Hind limbs extended caudally (observe from the side) |  |
|  | Top of the head below the line of spinal column (if not eating) |  |
|  | (0) All of the above-described behaviours are absent |  |
|  | (1) Presence of 1 of the above-described behaviours |  |
|  | (2) Presence of 2 or more of the above-described behaviours |  |
| **Posture when lying down** | Ventral recumbency with full or partial extension of one or both hind limbs |  |
|  | Head on/close to the ground |  |
|  | Extending the neck and body forward when in ventral recumbency |  |
|  | (0) All of the above behaviours are absent |  |
|  | (1) Presence of 1 of the above-described behaviours |  |
|  | (2) Presence of 2 or more of the above-described behaviours |  |
| **Miscellaneous behaviours 1** | Groaning |  |
|  | Attention towards the painful area |  |
|  | Licking the surgical wound |  |
|  | (0) All of the above behaviours are absent |  |
|  | (1) Presence of 1 of the above-described behaviours |  |
|  | (2) Presence of 2 or more of the above-described behaviours |  |
| **Miscellaneous behaviours 2** | Lambs’ ears, ears rotated back and the pinna facing down |  |
|  | Tense expression/strained appearance, furrows above the eyes and puckers above the nostrils |  |
|  | Wagging the tail abruptly and repeatedly |  |
|  | (0) All of the above behaviours are absent |  |
|  | (1) Presence of 1 of the above-described behaviours |  |
|  | (2) Presence of 2 or more of the above-described behaviours |  |
| **Limb movement/condition** | Lifting one foot of the ground |  |
|  | Kicking/foot stamping |  |
|  | Restlessness (pacing) |  |
|  | Weight shifting |  |
|  | (0) All of the above-described behaviours are absent |  |
|  | (1) Presence of 1 of the above-described behaviours |  |
|  | (2) Presence of 2 or more of the above-described behaviours |  |
| **Interactive behaviour with the environment** | (0) Active and attentive to environmental stimuli. When near other animals, can interact with and/or accompany the group |  |
|  | (1) Apathetic, interacting little when stimulated. When near other animals might remain close to them |  |
|  | (2) Apathetic; not reacting to environmental stimuli. When near other animals may be isolated or not accompany them |  |
| **Response to approach**  ***(if the animal is lying down or standing still stimulate it with clapping hands)*** | (0) Animal’s head up, ears forward, or may interrupt briefly ongoing activity (grooming, ruminating, etc.) |  |
|  | (1) Animal’s ears not forward, orients by moving head in the direction of the observer |  |
|  | (2) Animal’s ears back, head low, does not orient head toward the observer clapping hands (no head movement) |  |
| **Activity and locomotion**  ***(if the animal is lying down or standing still stimulate it with clapping hands)*** | (0) Moving normally. Walking with no obviously abnormal gait, or relaxed in ventral recumbency position (resting quietly), or standing still easily or eating or ruminating |  |
|  | (1) Walking with restriction, hunched back when moving or short steps. May be agitated (constant changes in weight-bearing) or laying restlessness (difficult to find a comfortable position) |  |
|  | (2) Reluctant to stand up, standing up with difficulty or not walking |  |
