# Supplementary material for: Bovine pain scale: A novel tool for pain assessment in cattle undergoing surgery in the hospital setting
Source: PLoS One. 2025 May 23;20(5):e0323710. doi: 10.1371/journal.pone.0323710 (PMC12101770; doi:10.1371/journal.pone.0323710)
Supplement: Attachment — Submitted filename: Reviewr comments.pdf [file pone.0323710.s009.pdf]

My suggestion based on PLOS ONE Standard:

1. **Research Originality:** The study presents a new method for measuring pain in hospitalized cattle: the Bovine Pain Scale (BPS). This uniqueness may be seen throughout the work, but it is particularly highlighted in the Introduction (lines 67–94) and Abstract (lines 39–62).
2. **Novelty of Results:** The results seem to be unique and haven't been published before, especially the scale's validation metrics (lines 202–227). Though there isn't a comparable all-inclusive tool, think about making it clear in the introduction.
3. **Technical and Analytical Standards:** Materials and Methods (lines 182–200) provide specifics on the statistical techniques, such as ROC curve analysis and intra-class correlation coefficients. There are problems with internal consistency in Results (lines 218–227), when scores fall below the permissible cutoff of 0.6. This calls for explanation or discussion of the consequences for implications for clinical applications.
4. **Data-Driven Conclusions:** The Discussion (lines 316–354) provides an effective summary of the conclusions, which are backed up by data. As may be observed in lines 344–348, the authors admit limits in construct validity and sensitivity.
5. **Language and Clarity:** The manuscript is generally well-written and clear. To facilitate wider accessibility, some sections, including the explanations of statistical analysis (lines 182–200), can benefit from simplification.
6. **Ethical Standards:** The Materials and Methods (lines 96–116) contain extensive documentation of ethical compliance, including the Institutional Animal Care and Use Committee's approval and measures to reduce animal suffering.
7. **Reporting criteria and Data Availability:** The validation procedure clearly complies with COSMIN (Consensus-based Standards for the selection of health Measurement Instruments) criteria (lines 97–102), and the Additional Information section makes clear that data is available.

Strengths:

- The manuscript addresses a critical gap in cattle pain management (lines 67–94).
- High inter- and intra-rater reliability (lines 210–216) demonstrates the scale's robustness.

- Strong correlation with visual analog scales (VAS) supports criterion validity (line 353).

Weaknesses and Suggestions

1. **Internal Consistency (Lines 218–227):** The internal consistency score is below the acceptable threshold. Consider discussing how this may affect clinical applications and whether modifications are planned to improve this metric.
2. **Potential Bias (Lines 152–160):** Surgical wounds were visible during video assessments, potentially biasing rater scores. Discuss strategies to minimize this issue in future studies.
3. **Sensitivity Issues (Lines 348–354):** The scale lacks sensitivity for certain pain indicators. Provide recommendations for refining these items to improve practical utility.
4. **Comparison with Existing Tools (Lines 83–94):** Expand comparisons with existing pain scales like UNESP-Botucatu Cattle Pain Scale (UCAPS) to emphasize the additional value of BPS.
5. **Simplification of Statistical Details (Lines 182–200):** Simplify complex explanations of statistical methods to enhance readability for a broader audience.

My recommendation:

Pending minor revisions addressing internal consistency, sensitivity, and clarity, the manuscript is recommended for publication in *PLOS ONE*.
