# Supplementary material for: Bovine pain scale: A novel tool for pain assessment in cattle undergoing surgery in the hospital setting
Source: PLoS One. 2025 May 23;20(5):e0323710. doi: 10.1371/journal.pone.0323710 (PMC12101770; doi:10.1371/journal.pone.0323710)
Supplement: Attachment — Submitted filename: Comments.docx [file pone.0323710.s010.docx]

Comments

Generally, it is a good study that enhances welfare management in animals. However, it should be clearly explained what has been done, and further clarification is needed. Some explanations are unclear as to whether they have been conducted or not. The second concern is that it is not exactly clear which study design has been used. In some parts, it appears experimental (randomized controlled trial), but it also seems to be a prospective cohort study. How compounding factors, such as environmental factors, were handled is not explained. This should be clearly addressed in the study design section. For a prospective study, the sample size is very small. How was the sample size calculated? The selection of control and exposed groups should also be clearly explained. In the results section, each output should be explained in detail; simply presenting it in table form is not enough. I observed wide gaps between raters. My detailed comments are given below.

1. **Line 45-46**: Videos were recorded before, 2 to 6 hours after surgery, 1 hour after the administration of analgesia and 24 hours after surgery. How long before the surgery were the videos taken?
2. Line 44, 47: Why do you choose the experimental to control group ratio 25:11? What effect size did you anticipated? It should be explained in the methods part
3. Financial disclosure: “This study was founded”, I think it should be funded
4. Line 79 may need to be cited
5. Line 93: hospital setting? need to be explained more, How modernized is? To which population are you going to generalize your results

- You should explain your population of interest.

6. Line 104: here it states 43 animals but in the abstract 36 study animals, there is mismatching

Have you considered stratification among age groups or sex groups? I would say pain among different age and sex groups could not be the same.

7. Line 106-109: Would you clarify a bit what soft tissue surgery is? Wound treatment, lameness and soft tissue surgery should be well differentiated. Lameness could occur because of soft tissue injury. Have you considered in accounting pain difference among surgery types?

- The eligibility and ineligibility criteria, inclusion and exclusion criteria, anesthesia protocol should be included as annex

8. Have you considered in accounting pain difference among surgery types?
9. Line 129: I would include P1 at least 12 hours before sedation, just to compare with the normal situation. The time before immediate sedation might involve some frustration.
10. Lines 131-132, What does pain group meaning? Are they experimental group?
11. Pain indicators should be explained in the methodology
12. Video raters: are they experienced in such analysis? In case they are not experienced their number is small
13. Line 184: Doesn't the latest version of RStudio exist?
14. Line 189; which non-parametric tests?
15. Table 1: I didn’t see heart rate and Blood pressure as pain indicators. Have you considered them?
16. The time for each video are not consistent. It could have impact during evaluation.
17. Line 195: The name of the table should be descriptive. Is these criteria’s after analgesics injection? Or during the overall surgery?
18. Results: Was training given to the raters? This should be explained in the methodology.

- This could be related to what each phrases (e.g. interactive behavior with the environment) mean?
- I see some wide gaps between raters; further training could help improve this.

19. I didn’t understand the relevance of Table 3 if it is not included in the analysis. Why are these variables not included in the analysis? You can also explain what the information in the table is descriptively.
20. The results are presented in tables with small explanations. Why? All of these should be explained in text form - what does each one mean? All the kappa values, CIs, ICCs between raters and of each variables should be explained and compared. The same should be done for all tables throughout the paper. Simply presenting them in tables is not enough
21. Rescue analgesia is, of course, needed when the pain worsens. However, what were the criteria for deciding whether to administer it? It seems subjective and could lead to bias
22. Table 6: I would be happy to see the true negative values of the variables in this table to estimate specificity. How do you explain this? I suggest including them in the text.
23. I woul like to see SE for all statistics. Correlation is no enough; adding SE for residuals and CI could add value
24. Line 290 (Table 8): This table needs to be described statistically in a way that is understandable to other readers. What does this analysis imply? What are the main outputs of this statistical analysis?
25. In this discussion, many efforts have been made, including addressing the limitations of the paper. This is good; however, despite these efforts, the quality of the paper is compromised. I would suggest further improvements to enhance the quality of the paper
26. Lines 341-343, Terms like 'good' or 'very good' may not be ideal for comparison; it would be better to use terms like 'higher,' 'better,' 'lower,' etc. as examples. You can choose more appropriate terms for your study.
