# Supplementary material for: Bovine pain scale: A novel tool for pain assessment in cattle undergoing surgery in the hospital setting
Source: PLoS One. 2025 May 23;20(5):e0323710. doi: 10.1371/journal.pone.0323710 (PMC12101770; doi:10.1371/journal.pone.0323710)
Supplement: Attachment — Submitted filename: 01 Response_PlosOne.docx [file pone.0323710.s012.docx]

Additional Editor Comments (if provided):

Considering the reviewers' suggestions, I ask that you make the necessary adjustments so that we can continue with the publication work.
Att

*Response: The authors appreciate the Reviewer´s time and effort spent reviewing this manuscript. Thank you for considering our manuscript for publication.*

**Reviewer #1:** The manuscript describes the evaluation of a bovine pain measurement system which was developed by including the experience with already published systems. The aim was the discrimination of painful and non-painful individuals. 25 animals were included in the pain group and 11 animals in the control group.
*Response: Thank you for your comments and review!*

The practical outcome of the study and its consequences are hard to evaluate due to two main points:
1. The results of the BPS study for both the pain group and the control group are only summarized in Supplemental Fig. S1 (a circle stands for one evaluation by one of the four raters?). This is an essential information to evaluate the study and, therefore, should not be classified as supplemental result:
*Response: This figure was added to the manuscript – figure 3. Line: 294.*
- Please provide the standard deviation of the high variation within the groups for all time points.
*Response: This analysis was not added because those variables are nonparametric, thus presenting it as median, first and third quartile is adequate. This was also done in other studies (Werneck et al. 2024, Luna 2020, Silva 2020).*
- What is the biological relevance of the difference between an average total score of 3-5 of 18 points in the control group and 5-6 of 18 points in the pain group (and by considering the high variation within the groups) for an individual which is now subsequently tested with the BPS?
*Response: A clinical study involving the development and validation of a pain scoring system does not precisely address the biological relevance of pain scores, especially when the study design does not differentiate mild, moderate and severe pain. However, the receiver operating characteristic (ROC) curve showed that scores of ≥ 5 of 18 better discriminates painful vs non-painful individuals, which true negatives and positives scoring lower or equal to 4 or higher or equal to 6, respectively. This was a clinical study, performed in a hospital environment. Clinical judgement must be used during pain assessment even using pain scoring tools. Our results showed that pain scores between 4-6 could fall into the diagnostic uncertainty zone. In this case, veterinarians should be aware that clinical judgment must be applied; animals may be reassessed as needed when presenting these scores. For example, analgesia may be administered if pain scores are 4 and the clinician feels that pain should be addressed.*

- How many of the animals of the pain group underwent analgetic treatment? In my opinion, the pain group has to be divided in two subgroups with/without analgetic treatment at least for the subsequent time points (or better for all time points?) for the statistical analyses of the results.
*Response: All animals received analgesic treatment (with NSAIDs or opioids) either pre-operatively or postoperatively. We did not have any animals without analgesic treatment; hence, unfortunately division into two subgroups is not possible and this procedure would not affect scale validity according to the statistical methods described in the manuscript.*
- Generally, it is not clear why all animals of the pain group are used together for the statistical analyses of the results. Presumably this may lead to a result which is highly specific for the study described. Does it make sense to divide the pain group according to the total score to carry out at least some of the statistical analyses for the new subgroups separately?
*Response: Please see our comment above. For the sake of scale validation regarding criterion validity, specificity and sensitivity, construct validity and responsiveness, pain scores should be pooled together and compared with controls and/or before the administration of analgesics using different types of pain and anesthetic/analgesics protocols. This approach mimics the clinical setting. Further studies may refine the Bovine Pain Scale by splitting groups according to type of surgery and drug protocols.*

2. For readers not involved in the use of pain scales in cattle, some essential information is not given in the manuscript and/or by only referring to other publications, e.g.:
- What is the rationale to use the chosen nine items of the BPS and, in addition, to use them with equal classification in view of the points given?
*Response: Pain produces several behavioral changes as represented in a composite pain scale. Equal weighting and associations of pain behaviors/items were tested using content validity, internal consistency, item-to-item correlation and multiple association with exploratory factor analysis. Therefore, each item of the scale has been tested to be included in the final version. Lines 124 to 132 explain the Bovine Pain Scale, each item is important for the assessment,* *scores 0 mean normal behavior and no pain, scores 1 and 2 represent pain related behaviors. Added to the text is an explanation. Lines: 127-128. Thanks for pointing this out. Our intention is in a future study to use artificial intelligence to weigh the importance of each behavior like performed for the Unesp-Botucatu Cattle Pain Scale (Trindade et al. 2024 -* *Ranking bovine pain-related behaviors using a logistic regression algorithm). This was included in discussion (lines: 465-466).*
- Results section, Tables 4 and 5: VAS: describe methodology and calculation.
*Response:* *Visual analog scale (VAS; 0-100 mm, where 1 = no pain and 100 = the worst pain imaginable) – added to both legends. This is described in Table 2 as follows: “For the sum of the BPS and VAS, the intraclass correlation coefficient (ICC; “icc{irr}”) two-way random effects model, type consistency multiple raters/measurements and its 95% CI based in 1,001 replications by the bootstrap method (“boot.ci{boot}”) was used. Interpretation of kw and ICC: very good 0.81 - 1.0; good 0.61 - 0.80; moderate 0.41 - 0.60; reasonable 0.21 - 0.4 and poor < 0.2 [21–23].”*
- Figures: Figure legends are lacking/incomplete.
*Response: They have been amended.*
- As much abbreviations are used, please provide a list of ALL abbreviations.
*Response: A list of abbreviations has been added as supplementary material. The abbreviations were described in full when they first appeared in the text.*

The aim of the study is to improve the recognition of pain in cattle. The Discussion section gives some hints for the comparison of the study results to already published results achieved with other pain scale tools. A more systematic comparison e.g. by including a table for this comparison would enhance the information content of the manuscript. A major point in this comparison is the description of the feasibility of the different scales.
*Response: More comparisons between the proposed scale versus UCAPS and CPS were included in Discussion (please see lines 346 and 362) and other comparisons had already been included (lines 371-373, 376-416). We appreciate the suggestion, however, the authors believe that the comparisons performed in the discussion are sufficient. The feasibility was not assessed with CPS or UCAPS. Hence, comparisons are not possible*

*Thank you,*
**Reviewer #2:** My suggestion based on PLOS ONE Standard:
Weaknesses and Suggestions
1. Internal Consistency (Lines 218–227): The internal consistency score is below the acceptable threshold. Consider discussing how this may affect clinical applications and whether modifications are planned to improve this metric.

*Response: Internal consistency was below the acceptable threshold due to a heterogeneous population undergoing different procedures and anesthetic protocols. We have provided additional information in the discussion (starting line 413).*

2. Potential Bias (Lines 152–160): Surgical wounds were visible during video assessments, potentially biasing rater scores. Discuss strategies to minimize this issue in future studies.

*Response: Strategies to address this potential bias have now been added.*

3. Sensitivity Issues (Lines 348–354): The scale lacks sensitivity for certain pain indicators. Provide recommendations for refining these items to improve practical utility.

*Response: This has been added to the discussion (lines 422-423). A recent study using a forest algorithm refined a pain scale and found the best pain behaviors set (Pivato et al 2025), this methodology should be used in the future to refine the bovine pain scale (lines 423-425).*

4. Comparison with Existing Tools (Lines 83–94): Expand comparisons with existing pain scales like UNESP-Botucatu Cattle Pain Scale (UCAPS) to emphasize the additional value of BPS.
*Response: Additional information has been added. However, comparisons among existing scales and the BPS are mostly provided in the discussion.*

5. Simplification of Statistical Details (Lines 182–200): Simplify complex explanations of statistical methods to enhance readability for a broader audience.

*Response: The authors followed the COSMIN guidelines (reference), which recommend statistical analyses to be provided in detail so the measurement properties of the instrument can be fully assessed. We provided a Table to facilitate reading and understanding. According to two systematic reviews, manuscripts on the developments of pain scoring instruments often have insufficient description of statistical analyses (Evangelista et al 2020, Tomacheuski et al 2023).*

My recommendation:
Pending minor revisions addressing internal consistency, sensitivity, and clarity, the manuscript is recommended for publication in PLOS ONE.
*Response: Thank you very much for your comments and review!*

 **My suggestion based on PLOS ONE Standard:**

1. **Research Originality:** The study presents a new method for measuring pain in hospitalized cattle: the Bovine Pain Scale (BPS). This uniqueness may be seen throughout the work, but it is particularly highlighted in the Introduction (lines 67–94) and Abstract (lines 39–62).
*Response: Thank you very much for your comments and review!*

2. **Novelty of Results:** The results seem to be unique and haven't been published before, especially the scale's validation metrics (lines 202–227). Though there isn't a comparable all-inclusive tool, think about making it clear in the introduction.
*Response: This has been added in the introduction. Lines: 91-93*

3. **Technical and Analytical Standards:** Materials and Methods (lines 182–200) provide specifics on the statistical techniques, such as ROC curve analysis and intra-class correlation coefficients. There are problems with internal consistency in Results (lines 218–227), when scores fall below the permissible cutoff of 0.6. This calls for explanation or discussion of the consequences for implications for clinical applications.
*Response: We have now addressed the issue of internal consistency in the discussion (starting line 413).*

4. **Data-Driven Conclusions:** The Discussion (lines 316–354) provides an effective summary of the conclusions, which are backed up by data. As may be observed in lines 344–348, the authors admit limits in construct validity and sensitivity.

*Response: Thank you*

5. **Language and Clarity:** The manuscript is generally well-written and clear. To facilitate wider accessibility, some sections, including the explanations of statistical analysis (lines 182–200), can benefit from simplification.
*Response: The authors followed the COSMIN guidelines (reference), which recommend statistical analyses to be provided in detail so the measurement properties of the instrument can be fully assessed. We provided a Table to facilitate reading and understanding. According to two systematic reviews, manuscripts on the developments of pain scoring instruments often have insufficient description of statistical analyses (Evangelista et al 2020, Tomacheuski et al 2023).*

6. **Ethical Standards:** The Materials and Methods (lines 96–116) contain extensive documentation of ethical compliance, including the Institutional Animal Care and Use Committee's approval and measures to reduce animal suffering.

*Response: Thank you*
7. **Reporting criteria and Data Availability:** The validation procedure clearly complies with COSMIN (Consensus-based Standards for the selection of health Measurement Instruments) criteria (lines 97–102), and the Additional Information section makes clear that data is available.
*Response: Thank you*

**Strengths:**

The manuscript addresses a critical gap in cattle pain management (lines 67–94).
High inter- and intra-rater reliability (lines 210–216) demonstrates the scale's robustness.
Strong correlation with visual analog scales (VAS) supports criterion validity (line 353).

Reviewer #3: Generally, it is a good study that enhances welfare management in animals. However, it should be clearly explained what has been done, and further clarification is needed. Some explanations are unclear as to whether they have been conducted or not. The second concern is that it is not exactly clear which study design has been used. In some parts, it appears experimental (randomized controlled trial), but it also seems to be a prospective cohort study. How compounding factors, such as environmental factors, were handled is not explained. This should be clearly addressed in the study design section. For a prospective study, the sample size is very small. How was the sample size calculated? The selection of control and exposed groups should also be clearly explained. In the results section, each output should be explained in detail; simply presenting it in table form is not enough. I observed wide gaps between raters. Find detailed comments in the attachment

*Response: Thank you very much for your comments and review!*

Comments

Generally, it is a good study that enhances welfare management in animals. However, it should be clearly explained what has been done, and further clarification is needed. Some explanations are unclear as to whether they have been conducted or not. The second concern is that it is not exactly clear which study design has been used. In some parts, it appears experimental (randomized controlled trial), but it also seems to be a prospective cohort study. How compounding factors, such as environmental factors, were handled is not explained. This should be clearly addressed in the study design section. For a prospective study, the sample size is very small. How was the sample size calculated? The selection of control and exposed groups should also be clearly explained. In the results section, each output should be explained in detail; simply presenting it in table form is not enough. I observed wide gaps between raters. My detailed comments are given below.

*Response: The study design was a prospective, observational clinical study as described in the first two lines of the M&M. A detailed description of the animal population has been described in the Methods. The Pain Group consisted of patients recruited at the CHUV (veterinary teaching hospital) with appropriate inclusion and exclusion criteria. The sample size was based on a previous study (Tomacheuski et al 2023) (lines 201-204), but the small sample size was due to the impact of COVID 19 (this is addressed in the manuscript). This study was performed at the CHUV and environment (single stalls) is described in the Methods with appropriate acclimation protocols to cameras and personnel, etc.* *The Control Group consisted of animals from the teaching colony that were deemed healthy based on history, physical examination and regular blood work. The table demonstrates the demographics of the pain group. The available videos can also provide additional views of the hospital facilities and how animals were assessed for pain.*

1. **Line 45-46**: Videos were recorded before, 2 to 6 hours after surgery, 1 hour after the administration of analgesia and 24 hours after surgery. How long before the surgery were the videos taken?

*Response: It is addressed in the lines 136-137 “P1, preoperatively, immediately prior to administration of sedatives”*

2. Line 44, 47: Why do you choose the experimental to control group ratio 25:11? What effect size did you anticipated? It should be explained in the methods part

*Response: A total of 25 animals were admitted and eligible to be included in the study during the clinical trial, which was affected by COVID-19. We used the total number of controls that were available for filming. The minimum sample size was calculated from a previous study (Tomacheuski et al 2023) (lines 198-201), but the COVID 19 impacted the study, as previously mentioned.*

Financial disclosure: “This study was founded”, I think it should be funded
*Response: Amended.*

3. Line 79 may need to be cited

*Response: Citation added.*

4. Line 93: hospital setting? need to be explained more, How modernized is? To which population are you going to generalize your results

- You should explain your population of interest. \

*Response: We have added information about the hospital setting: “a referral hospital that receives over 1000 farm animals every year. Its modern hospital is the clinical center of reference of Quebec, Canada offering cutting-edge diagnosis and treatment of farm animals with advanced equipment and surgery as well as training programs”.*

Line 104: here it states 43 animals but in the abstract 36 study animals, there is mismatching

Have you considered stratification among age groups or sex groups? I would say pain among different age and sex groups could not be the same.

*Response: On lines 220-221 it is stated “Seven animals were excluded from Phase 2 of study because their videos were used in Phase 1.” That is why only 36 animals were included in Phase 2.*

5. Line 106-109: Would you clarify a bit what soft tissue surgery is? Wound treatment, lameness and soft tissue surgery should be well differentiated. Lameness could occur because of soft tissue injury. Have you considered in accounting pain difference among surgery types?

- The eligibility and ineligibility criteria, inclusion and exclusion criteria, anesthesia protocol should be included as annex

*Response: Table 3 describes the surgical procedures included in the study. We do not believe there should be a need to provide further clarification to the audience on which procedures were soft tissue or orthopedic as we did not have any cases of lameness or wound treatment. Soft tissue surgery in cattle encompasses procedures addressing issues with internal organs, body wall, masses, tumors, hernias, and other conditions, excluding those related to bones, joints, muscles, or the nervous system. Cases were unbalanced to carry out any analyses that differentiate the procedures. Eligibility and ineligibility criteria, inclusion and exclusion criteria are described in lines: 110-113. The anesthesia protocols for each animal have been added as supplementary material.*

6. Have you considered in accounting pain difference among surgery types?

*Response: As previously answered the number of cases were unbalanced to carry any robust analyses. For scale validation, pain scores of treated animals should be pooled together and compared to controls or before and after administering analgesics.*

7. Line 129: I would include P1 at least 12 hours before sedation, just to compare with the normal situation. The time before immediate sedation might involve some frustration.

*Response: The authors will take this suggestion into account for a potential future study.*

8. Lines 131-132, What does pain group meaning? Are they experimental group?

*Response: The Pain Group consisted of patients admitted to the CHUV requiring any type of soft tissue or orthopedic surgery as described in the Methods. There is no experimental group. Animals were clinical patients admitted to the CHUV. We are avoiding the terminology “experimental” to avoid confusion as this was a prospective, observational, controlled clinical trial. Lines: 111-113*

9. Pain indicators should be explained in the methodology

*Response: The pain behaviours cited in the Bovine Pain Scale are examples of pain indicators in cattle. Table 1. Full videos are provided so it is difficult to provide even more details.*

10. Video raters: are they experienced in such analysis? In case they are not experienced their number is small

*Response: All raters were experienced veterinarians with training in pain assessment. This has been amended. According to Streiner et al 2015 and the COSMIN guidelines, a minimum of 3 raters is recommended, and our study presented 4 raters.*

11. Line 184: Doesn't the latest version of RStudio exist?

*Response: This was the version used for the analysis.*

12. Line 189; which non-parametric tests?

*Response: Table 2 explains in detail all the nonparametric tests carried out in the analysis.*

13. Table 1: I didn’t see heart rate and Blood pressure as pain indicators. Have you considered them?

*Response: Even though heart rate and blood pressure could be related to pain, they are strongly biased by neurohumoral input (cortisol release), fear, anxiety or dehydration/hypovolemia. Pain behaviors are the best indicators to assess pain in cattle.*

14. The time for each video are not consistent. It could have impact during evaluation.

*Response: Timing of video analysis is adequate for the purpose of scale development and validation. It must involve the same individuals before and after surgery and before and after the administration of analgesia, and allow comparisons between controls and treated animals overtime – please see the book by David Streiner on “Health Measurement Scales”. However, this is discussed as a limitation on lines 456-458.*

15. Line 195: The name of the table should be descriptive. Is these criteria’s after analgesics injection? Or during the overall surgery?

*Response: This has been amended. All animals received analgesic treatment.*

16. Results: Was training given to the raters? This should be explained in the methodology.

- This could be related to what each phrases (e.g. interactive behavior with the environment) mean?

*Response: All raters received training (lines 162-167).*

- I see some wide gaps between raters; further training could help improve this.

*Response: All raters received training and were veterinarians with experience on pain assessment. Individual variability is normal and it is accepted in clinical trials regarding pain scoring. We made every possible attempt to minimize bias in the context of the limitations of the study.*

17. I didn’t understand the relevance of Table 3 if it is not included in the analysis. Why are these variables not included in the analysis? You can also explain what the information in the table is descriptively.

*Response: Table 3 provides demographic data of our population in the Pain Group. It would be awkward to report our results without reporting clinical context, patient population and type of surgery. Sample size does not allow subgroup comparisons (sex, surgery type or type of stable).*

18. The results are presented in tables with small explanations. Why? All of these should be explained in text form - what does each one mean? All the kappa values, CIs, ICCs between raters and of each variables should be explained and compared. The same should be done for all tables throughout the paper. Simply presenting them in tables is not enough

*Response: Every table is cited and explained in the text with the most relevant results. We carefully avoided overlapping information in the manuscript vs tables.*

19. Rescue analgesia is, of course, needed when the pain worsens. However, what were the criteria for deciding whether to administer it? It seems subjective and could lead to bias

*Response: Similar methodology has been used in previous studies (Luna et al 2020, Silva et al 2021, Tomacheuski et al 2023, Werneck et al 2024) and it is widely accepted during scale development. This approach is used to calculate the ROC curve and cut-off point using the clinical judgment of an experienced veterinarian after watching the video. However, during real-time assessment, analgesia was administered as follows: “At P3, rescue analgesia was administered if pain scores were ≥ 5 of 10 according to the UCAPS with the agreement of the attending clinician”.*

20. Table 6: I would be happy to see the true negative values of the variables in this table to estimate specificity. How do you explain this? I suggest including them in the text.

*Response: Corrected to Table 7. True negative values are the same as specificity. For the context of scale validation and the multiple measurement properties reported, the information provided about specificity and specificity is adequate for each item of the scale indicating which ones had over 70%. This should suffice for the audience who has an interest in the clinical relevance of the pain scale.*

21. I woul like to see SE for all statistics. Correlation is no enough; adding SE for residuals and CI could add value

*Response: The results have been described using rigorous standards by the COSMIN guidelines and Health Measurement Scale properties (Streiner et al 2015). Additionally, the journal does not require SE reporting.*

22. Line 290 (Table 8): This table needs to be described statistically in a way that is understandable to other readers. What does this analysis imply? What are the main outputs of this statistical analysis?

*Response: Corrected to Table 9. All statistical analysis is described in detail in Table 2. Responsiveness is the ability to detect changes over time. This is a well-known term for the development and validation of pain scales (Brondani et al 2011, Luna et al 2020, Silva et al 2021, Belli et al 2021, Tomacheuski et al 2023, Werneck et al 2024).*

23. In this discussion, many efforts have been made, including addressing the limitations of the paper. This is good; however, despite these efforts, the quality of the paper is compromised. I would suggest further improvements to enhance the quality of the paper

*Response: We have addressed every comment provided by the three reviewers, including yours. The discussion has been amended.*

24. Lines 341-343, Terms like 'good' or 'very good' may not be ideal for comparison; it would be better to use terms like 'higher,' 'better,' 'lower,' etc. as examples. You can choose more appropriate terms for your study.

*Response: These terms are used by systematic reviews and scale development and validation (Tomacheuski et al 2023, Evangelista et al 2022, Luna et al 2020, Silva et al 2020, Belli et al 2021, Werneck et al 2024, Prinsen et al 2018, Streiner et al 2015). They are all related to how statistical analyses are reported. References regarding these classifications have been included throughout the manuscript.*
